# Supplementary material for: Time Trends of Crohn’s Disease in Catalonia from 2011 to 2017. Increasing Use of Biologics Correlates with a Reduced Need for Surgery
Source: J Clin Med. 2020 Sep 8;9(9):2896. doi: 10.3390/jcm9092896 (PMC7563515; doi:10.3390/jcm9092896)
Supplement: Supplementary file 1 [file jcm-09-02896-s001.pdf]

Supplementary Files

Supplementary Table S1. List of ICD-9 diagnoses for CD.

**555**	**Crohn's Disease**
5550	Small bowel regional enteritis. Crohn's disease of duodenum, ileum, jejunum; ileitis: regional, segmental, terminal
5551	Large bowel regional enteritis. Crohn's disease of colon, large bowel, rectum; granulomatous, regional or transmural colitis: granulomatous, regional, transmural
5552	Small and large bowel regional enteritis. Ileo-colitis regional
5559	Regional enteritis unspecified. Crohn's disease NOS, regional enteritis NOS

Supplementary Table S2. List of drugs used in CD and ATC codes.

**Non-Biological Immunosuppressive Treatment**	
L04AX01	Azathioprine
L01BB02	Mercaptopurine
L04AX03	Methotrexate
L04AD01	Cyclosporine
**Salicylates**	
A07EC02	Mesalazine
A07EC01	Sulfasalazine
**Biological Treatments**	
L04AB02	Infliximab, Infliximab biosimilar
L04AB04	Adalimumab
L04AB06	Golimumab
L04AA33	Vedolizumab
L04AC05	Ustekinumab
**Systemic Corticosteroids**	
H02AB07	Prednisone
H02AB06	Prednisolone
H02AB04	Methylprednisolone
H02AB09	Hydrocortisone
H02AB13	Deflazacort
H02AB08	Triamcinolone
H02AB02	Dexamethasone
H02AB08	Triamcinolone
H02AB01	Betamethasone
**Topical Corticosteroids**	
A07EA07	Beclomethasone dipropionate
A07EA06	Budesonide
C05AA12	Triamcinolone diacetate

Supplementary Table S3. ICD-9 codes for surgical procedures.

**Code**	**Description**
**Ostomies**	
46.1	Colostomy
46.10	Colostomy, non specified
46.11	Temporal colostomy
46.13	Permanent colostomy
46.14	Delayed colostomy overage
46.2	Ileostomy

46.20	Ileostomy, non-specified
46.21	Temporal ileostomy
46.22	Continent ileostomy
46.23	Other permanent ileostomy
46.24	Delayed ileostomy overage
46.3	Other enterostomies
46.31	Other delayed enterostomies overage
46.4	Intestinal stoma review
46.40	Intestinal stoma review, non specified
46.41	Small bowel intestinal stoma review
46.42	Hernia repair pericolostomy
46.43	Other colonic stoma review
46.5	Bowel stoma closure
46.50	Bowel stoma closure, non specified
46.51	Small bowel stoma closure
46.52	Colostomy or cecostomy closure
**Bowel Resection**	
45.6	Other small bowel resection
45.61	Segmentary small bowel resection / traumatic lesions
45.62	Other partial resections of the small intestine
45.63	Total resection of the small bowel
17.3	Laparoscopic partial excision of colon
17.31	Multiple laparoscopic segmental resection of the colon
17.32	Laparoscopic cecectomy
17.33	Laparoscopic right hemicolectomy
17.34	Laparoscopic resection of the transversal colon
17.35	Laparoscopic left hemicolectomy
17.36	Laparoscopic sigmoidectomy
17.39	Others laparoscopic segmental resection of the colon
45.7	Total colon resection
45.71	Multiple laparotomy segmental resection of the colon
45.72	Laparotomy cecostomy
45.73	Laparotomy right hemicolectomy
45.74	Laparotomy resection of the transversal colon
45.75	Laparotomy left hemicolectomy
45.76	Laparotomy sigmoidectomy
45.79	Others laparotomy resection of the colon
45.8	Total colectomy
45.81	Total laparoscopic colectomy
45.82	Total laparotomy colectomy
45.83	Other non-specified colectomy
48.4	Rectal resection
48.40	Rectal resection non specified
48.41	Submucosal rectal resection (Soave)
48.42	Laparoscopic rectal resection
48.43	Laparotomy rectal resection
48.49	Other rectal resections / Swenson proctectomy
48.5	Rectal abdominoperineal resection
48.50	Rectal abdominoperineal resection, non-specified
48.51	Laparoscopic rectal abdominoperineal resection
48.52	Laparotomy rectal abdominoperineal resection
48.59	Other rectal abdominoperineal resection

48.6	Other rectal resections
48.61	Rectosigmoidectomy trans sacrum
48.62	Anterior rectal resection with simultaneous colostomy
48.63	Other anterior rectal resection
48.64	Posterior rectal resection
48.65	Duhamel rectal resection
48.69	Other rectal resections; partial proctectomy, non-specified resection
	**Other Therapeutic Procedures**
45.52	Colon segment isolation; interposition
45.9	Intestinal anastomosis
45.90	Intestinal anastomosis, non-specified
45.91	Small bowel to small bowel anastomosis
45.92	Small bowel to rectal sleeve anastomosis (Hampton)
45.93	Small bowel to colon anastomosis
45.94	Colon to colon anastomosis
45.95	Small bowel to anus anastomosis
46.0	Intestinal externalization
46.01	Small bowel externalization, ileostomy
46.02	Resection of externalized small bowel
46.03	Colon externalization (first step Mikulicz procedure)
46.04	Resection of externalized colon (second step Mikulicz procedure)
49.01	Perianal abscess incision
49.02	Other perianal abscess incision
49.04	Other perianal tissue excision
49.1	Anal fistula incision or excision
49.11	Anal fistulotomy
49.12	Anal fistulectomy
49.3	Local excision / destruction of anal tissue
49.39	Other excision / destruction of anal tissue

Supplementary Table S4. Use of drugs in CD from 2011 to 2017. Absolute number and percentage of CD patients treated.

	General Population	CD Patients	Salicylates		Corticosteroids		Non-Biological Immunosuppressive Treatments		Biological Treatments	
			Number	Percentage	Number	Percentage	Number	Percentage	Number	Percentage
2011	7,515,398	9187	2647	28.8%	1455	15.8%	3022	32.9%	1381	15.0%
2012	7,478,968	10,211	2762	27.0%	1526	14.9%	3315	32.5%	1645	16.1%
2013	7,433,894	11,439	2825	24.7%	1620	14.2%	3529	30.9%	1852	16.2%
2014	7,424,754	12,602	2905	23.1%	1767	14.0%	3800	30.2%	2038	16.2%
2015	7,448,332	13,768	2905	21.1%	1909	13.9%	4073	29.6%	2311	16.8%
2016	7,496,276	14,902	2828	19.0%	2057	13.8%	4353	29.2%	2674	17.9%
2017	7,543,825	16,528	2831	17.1%	2264	13.7%	4893	29.6%	3087	18.7%

Supplementary Table S5. Biologic treatment. Percentage are calculated by dividing the number of patients receiving the drug by the total number of patients with CD.

		Biologic Treatment									
	CD Patients	Infliximab		Adalimumab		Golimumab		Vedolizumab		Ustekinumab	
		Number	Percentage	Number	Percentage	Number	Percentage	Number	Percentage	Number	Percentage
2011	9187	754	8.2%	674	7.3%	1	0.0%	0	0.0%	4	0.0%
2012	10,211	876	8.6%	821	8.0%	4	0.0%	0	0.0%	6	0.1%
2013	11,439	942	8.2%	956	8.4%	7	0.1%	0	0.0%	9	0.1%
2014	12,602	1022	8.1%	1075	8.5%	14	0.1%	0	0.0%	18	0.1%
2015	13,768	1119	8.1%	1218	8.8%	21	0.2%	25	0.2%	45	0.3%
2016	14,902	1258	8.4%	1364	9.2%	29	0.2%	127	0.9%	56	0.4%
2017	16,528	1358	8.2%	1604	9.7%	24	0.1%	174	1.1%	127	0.8%

Supplementary Table S6. Surgical and diagnostic procedures in CD from 2011 to 2017. Absolute number and rate per 1000 patients/year.

	General Population	CD Patients	Ostomies		Resections		Other Surgical Procedures	
			Number	Rate Per 1000 Patients/Year	Number	Rate Per 1000 Patients/Year	Number	Rate Per 1000 Patients/Year
2011	7,515,398	9187	113	13.2	206	24.1	182	21.3
2012	7,478,968	10,211	102	10.7	230	24.1	193	20.2
2013	7,433,894	11,439	112	10.5	274	25.7	222	20.8
2014	7,424,754	12,602	119	10.1	279	23.6	211	17.9
2015	7,448,332	13,768	130	10.0	274	21.2	229	17.7
2016	7,496,276	14,902	111	7.9	248	17.6	210	14.9
2017	7,543,825	16,528	152	9.8	279	18.0	244	15.7

Supplementary Table S7. Pearson correlation coefficient (*p* value) between pharmacological treatment and surgical procedures and CD- related hospitalization.

	Ostomies	Resections	Other Surgical Procedures	CD-Related Hospitalization
Salicylates	0.798 (0.031)	0.863 (0.012)	0.934 (0.002)	0.924 (0.003)
Systemic Corticosteroids	0.863 (0.012)	0.572 (0.179)	0.776 (0.040)	0.875 (0.010)
Non-Biological Immunosuppressive Treatments	0.840 (0.018)	0.706 (0.076)	0.868 (0.011)	0.934 (0.002)
Biological Agents	−0.778 (0.039)	−0.887 (0.008)	−0.892 (0.007)	−0.793 (0.033)

Supplementary Table S8. Hospitalization. Absolute number and rate per 1000 patients/year.

	CD Patients	Hospitalization Due to Any Cause		Unscheduled Hospitalization Due to Any Cause		Hospitalization for CD		Hospitalization for Infectious Disease		Hospitalization for Neoplasm	
		Number	Rate	Number	Rate	Number	Rate	Number	Rate	Number	Rate
2011	9187	2334	272.8	1316	153.8	793	92.7	93	10.9	152	17.8
2012	10,211	2525	264.2	1420	148.6	850	88.9	117	12.2	159	16.6
2013	11,439	2923	273.7	1650	154.5	930	87.1	142	13.3	181	16.9
2014	12,602	3305	279.7	1868	158.1	919	77.8	147	12.4	225	19.0
2015	13,768	3484	269.1	1958	151.2	924	71.4	186	14.4	237	18.3
2016	14,902	4190	298.1	2373	168.8	1060	75.4	220	15.7	309	22.0
2017	16,528	4520	291.6	2560	165.1	1119	72.2	249	16.1	295	19.0

Supplementary Table S9. Hospitalization. Absolute number and rate per 100,000 inhabitants/year.

	General Population	Hospitalization Due to Any Cause		CD-Related Hospitalization	
		Number	Rate	Number	Rate
2011	7,515,398	2334	31.1	793	10.6
2012	7,478,968	2525	33.8	850	11.4
2013	7,433,894	2923	39.3	930	12.5
2014	7,424,754	3305	44.5	919	12.4
2015	7,448,332	3484	46.8	924	12.4
2016	7,496,276	4190	55.9	1060	14.1
2017	7,543,825	4520	59.9	1119	14.8

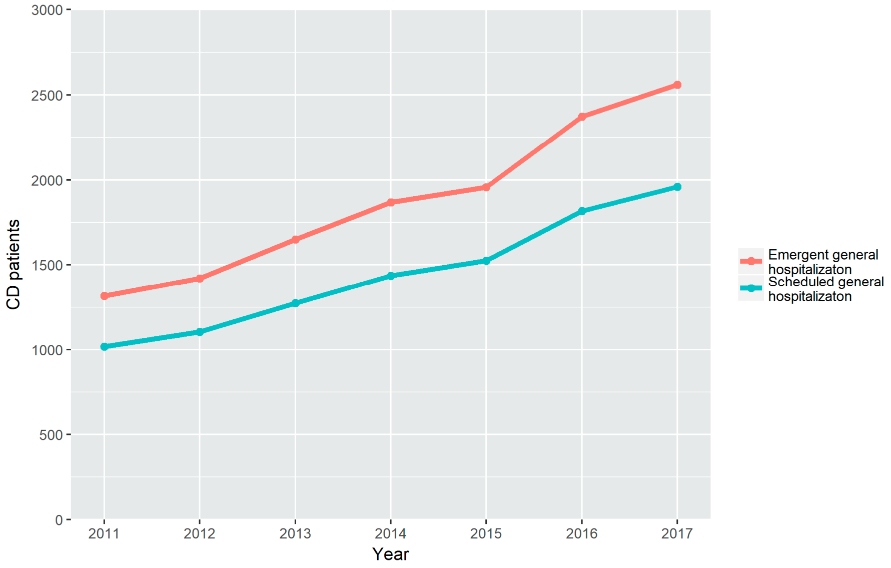

Supplementary Figure S1. Total CD patients hospitalized due to any cause either for scheduled and for emergency admissions. Rate per 1000 inhabitants/year.

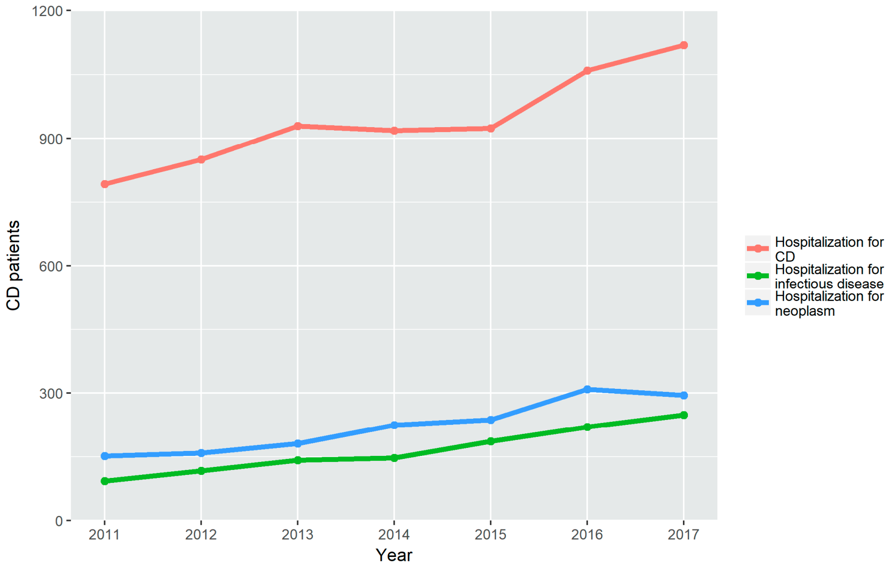

Supplementary Figure S2. CD patients hospitalized for CD cause, infections and neoplasm.
